# Candida parapsilosis: An Unusual Cause of Infective Endocarditis

**DOI:** 10.7759/cureus.3553

**Published:** 2018-11-06

**Authors:** Akriti G Jain, Jian Guan, Jason D'Souza

**Affiliations:** 1 Internal Medicine, Florida Hospital, Orlando, USA; 2 Cardiology, University of Missouri / St. Luke's Health System, Kansas City, USA

**Keywords:** fungal endocarditis, infectious endocarditis

## Abstract

Fungal endocarditis (FE) is a rare form of infectious endocarditis caused by fungi. Herein, we report a case of fungal pacemaker lead endocarditis. FE, especially in the setting of in-vivo cardiac devices, is on the rise. A high index of suspicion is required for its management that involves a combination of medical and surgical therapy.

## Introduction

Fungal endocarditis (FE) is a relatively uncommon form of endocarditis, accounting for only 1.3% to 6% of all cases of infectious endocarditis [1]. Although *Candida albicans* is a well-known cause of FE, the predominant subtype of non-albicans species causing endocarditis is *Candida parapsilosis* (*C. parapsilosis*). FE has been reported to have increased in incidence during the last 2 decades, accounting for almost 2% to 5% of infectious endocarditis cases [2-3]. In addition, the fungal involvement of pacemakers is also on the rise due to the increasing use of such intracardiac devices [4]. We hereby report a case of fungal pacemaker lead endocarditis and also highlight the challenges posed in treating this patient.

## Case presentation

**Figure 1:**
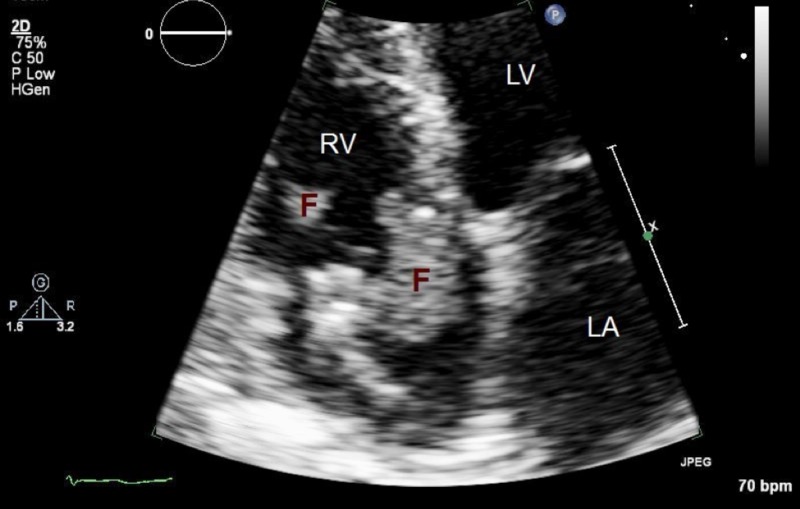
Trans-thoracic echocardiogram TTE showing a large 2 x 2-cm sessile mass (F) attached to the tricuspid valve which prolapsed into the right atrium during systole. TTE: trans-thoracic echocardiogram

A 60-year-old woman with a history of diabetes, ischemic cardiomyopathy (ejection fraction 30% to 35%) with implantable cardioverter-defibrillator (AICD) and pancytopenia of unclear etiology presented to the hospital with a three-day history of fever and altered mental status. On admission, the patient was febrile with a temperature of 102.6 °F, tachycardic with a heart rate of 102 beats per minute, respiratory rate of 16 breaths/minute and blood pressure of 160/87 mmHg. Her cardiac examination as well as the examination of her peripheral extremities was unremarkable. Laboratory findings revealed pancytopenia (white blood cells: 1.71 x 10^3^/μL, hemoglobin 6.6 g/dL and platelets 88 x 10^3^/μL). Imaging studies including chest X-ray, non-contrasted computed tomography (CT) head and CT abdomen were mostly unremarkable, except for mild splenomegaly. However, two of her blood cultures were positive for *C. parapsilosis*. Given that she was immunocompromised and had an indwelling prosthetic device in place, the patient was started on intravenous (IV) micafungin, and an echocardiogram was performed. The imaging revealed a large 2 x 2-cm sessile mass attached to the tricuspid valve that prolapsed into the right atrium during systole (Figure 1). The patient underwent removal of the AICD, coronary sinus lead and the right atrial lead under fluoroscopic guidance. Post-procedure trans-esophageal echocardiogram (TEE) at this point still demonstrated a mobile 1-cm vegetation on the tricuspid valve. Hence, it was concluded that our patient has an infection involving not only the AICD lead but also the native tricuspid valve since even after removal of the lead, there was a persistent vegetation attached to the tricuspid valve. It was presumed that the small vegetation that was still found to be remaining would improve with medical therapy. IV micafungin was continued after the procedure, and blood cultures were followed. One of the new blood cultures continued to grow *C. parapsilosis*. Repeat trans-thoracic echocardiogram (TTE) a few days later still demonstrated a mobile 2 x 2-cm vegetation on the right atrial side of the tricuspid valve. As the patient failed to clear the fungemia, a repeat surgery was undertaken with sternotomy and removal of multiple tricuspid valve vegetations, resection of an infected papillary muscle with reduction and remodeling annuloplasty of the tricuspid valve. The pathology report confirmed numerous fungal yeasts and pseudohyphal forms on Gram stain. The patient’s antifungal regimen was also modified to flucytosine and amphotericin B. The patient continued to improve from the cardiac standpoint; blood cultures after the second surgery were reported to be negative.

## Discussion

*C. parapsilosis* was earlier considered to be a non-pathogenic strain until 1940 when it was identified to be the causative agent of a fatal case of endocarditis in an intravenous drug user [5]. Some of the common predisposing factors for *C. parapsilosis* include the prosthetic valves (57.4%), IV drug use (IVDU; 20%), IV parenteral nutrition (6.9%), abdominal surgery (6.9%), immunosuppression (6.4%), treatment with broad-spectrum antibiotics (5.6%) and previous valvular disease (4.8%) [1]. In patients who underwent placement of pacemakers, the overall incidence of infections was observed to be 0% to 12.6% with rates ranging from 0.13% to 19.9% for permanent pacemakers and 0.2% to 7.2% for ICDs [6-7]. *Staphylococci* are the most common pathogens isolated in ICD-related endocarditis (60% to 80%), and fungal endocarditis accounts for 1% to 6%. FE in the absence of in-vivo cardiac devices has been extensively studied. The mortality rate was observed to be as high as 41.7% [1].

The clinical features of Candida* *endocarditis are non-specific [8]. Peripheral embolic and/or hemorrhagic events most commonly in the cerebral and lower limb territories were seen in 43.8% patients in a review on 72 patients with *C.* *parapsilosis* endocarditis by Garzoni et al. [1]. Our patient presented with fever and an altered mental status and did not have any cardiac findings or embolic phenomenon. Most patients have risk factors for invasive candidiasis. Our patient was known to have a history of diabetes mellitus, compromised immune status due to pancytopenia, splenomegaly and a cardiac device in situ but lacked the traditional risk factors such as IV catheter, IVDU and use of broad-spectrum antibiotics. Persistent candidemia in an immunocompromised host with an intra-cardiac device as seen in our case should be evaluated with early TTE and/or TEE as recommended by the European Society of Clinical Microbiology and Infectious Disease (ESCMID) guidelines for non-neutropenic adults with candidemia [8-9].

Per ESCMID guidelines, the treatment of native valve endocarditis should include surgery within one week, while in the presence of a prosthetic valve, earlier surgery is beneficial. They suggest an antifungal regimen consisting of amphotericin B +/- flucytosine [9]. The Infectious Diseases Society of America (IDSA) 2009 guidelines were similar, but also added the choice of using an echinocandin followed by a step-down therapy to fluconazole in patients who are stable and have negative blood cultures [8]. But both guidelines strongly recommend the removal of the surgical device. There are a few case reports showing the complete treatment of pacemaker Candida endocarditis with anti-fungals without surgical explantation [4]. Long-term suppressive fluconazole therapy has also been described, but the duration of treatment remains controversial [10-11]. Silva-Pinto et al. reported the use of amphotericin B in 65% cases, azoles in 35% and echinocandins in only 7% of all reported *C. parapsilosis* endocarditis (84) published till 2015 [3], thereby showing the lack of consensus on the most suited medical treatment and duration of therapy. Despite the use of a combination of medical and surgical therapy mortality rate of *C. parapsilosis* endocarditis remains as high as 40% [3].

In a prospective observational cohort study on 70 patients with Candida endocarditis, Arnold et al. reported older age, heart failure (HF) at baseline, persistent candidemia, nosocomial acquisition, HF as a complication and intracardiac abscess to be associated with higher mortality. They also found that the mortality was not affected by the use of surgical therapy or the choice of anti-fungal agents [12]. Our patient underwent a combination of surgical and medical management. Our patient underwent two surgeries, the first one being the extraction of the AICD and its lead under fluoroscopic guidance. Two weeks after the first surgery, even though the patient was on IV micafungin, the blood cultures continued to grow *C. parapsilosis*, which led to open heart surgery with the removal of tricuspid vegetation and annuloplasty. After the second surgery, the patients' antifungal regimen was also changed to flucytosine with amphotericin B. Blood cultures obtained after the second surgery were negative, and the patient continued to improve from the cardiovascular perspective. Hence, our case raises significant learning points about the timing and type of surgical approach and the choice of medical management. Some studies have shown that echinocandins are not as effective for *C. parapsilosis* [10], but patient factors such as renal failure may drive us to use echinocandins over amphotericin B.

## Conclusions

FE, especially in the setting of in-vivo cardiac devices, is on the rise. A high index of suspicion is required for diagnosis, and the treatment usually involves a combination of medical and surgical therapies.
